# Melatonin Regulates the Periodic Growth of Cashmere by Upregulating the Expression of *Wnt10b* and β*-catenin* in Inner Mongolia Cashmere Goats

**DOI:** 10.3389/fgene.2021.665834

**Published:** 2021-07-09

**Authors:** Junyang Liu, Qing Mu, Zhihong Liu, Yan Wang, Jiasen Liu, Zixian Wu, Wendian Gong, Zeyu Lu, Feifei Zhao, Yanjun Zhang, Ruijun Wang, Rui Su, Jinquan Li, Hongmei Xiao, Yanhong Zhao

**Affiliations:** ^1^College of Animal Science, Inner Mongolia Agricultural University, Hohhot, China; ^2^Laboratory of Animal Genetic, Breeding and Reproduction, Hohhot, China; ^3^Department of Inner Mongolia Academy of Agricultural Animal & Husbandry Sciences, Hohhot, China; ^4^College of Life Science, Inner Mongolia Agricultural University, Hohhot, China

**Keywords:** melatonin, transcriptome sequencing, differently expressed genes, cashmere goats skin, *Wnt*/β*-catenin*

## Abstract

Secondary hair follicle growth in cashmere goats has seasonal cycle changes, and melatonin (MT) has a regulatory effect on the cashmere growth cycle. In this study, the growth length of cashmere was measured by implanting MT in live cashmere goats. The results indicated that the continuous implantation of MT promoted cashmere to enter the anagen 2 months earlier and induce secondary hair follicle development. HE staining of skin tissues showed that the number of secondary hair follicles in the MT-implanted goats was significantly higher than that in the control goats (*P* < 0.05). Transcriptome sequencing of the skin tissue of cashmere goats was used to identify differentially expressed genes: 532 in February, 641 in October, and 305 in December. Fluorescence quantitative PCR and Western blotting results showed that MT had a significant effect on the expression of *Wnt10b*, β*-catenin*, and proteins in the skin tissue of Inner Mongolia cashmere goats. This finding suggested that MT alters the cycle of secondary hair follicle development by changing the expression of related genes. This research lays the foundation for further study on the mechanism by which MT regulates cashmere growth.

## Introduction

In China, goat breeds can be divided into dairy type, cashmere type, and meat type according to their economic uses (Watkins and Buxton, 1992). Cashmere goats are precious livestock resources for the production of natural fiber with high quality (Liu et al., 2016; Su et al., 2018). Cashmere and wool are important components of goat hairs. A study on the characteristics of cashmere goat coats reported that the complex shape and structure of hair follicles control hair growth. In mammals, hair follicles attached to the skin structure are skin micro-organs (Cetera et al., 2017) and develop through the interaction between epithelial and dermal cells. Regeneration is the most prominent characteristic of hair follicles (Zawilska et al., 1995; Tan et al., 1999). Follicles are divided into primary hair follicles and secondary hair follicles according to different developmental stages. Wool is developed by primary hair follicles, while cashmere is derived from secondary hair follicles of the skin (Zhu et al., 2013). Adult animals typically have one cashmere growth cycle in a year, with growth peaking in summer and slowing down in winter, and natural shedding occurring in spring (Mcdonald et al., 1987; Norton and Klören, 1995). In contrast, the growth of secondary hair follicles in adult cashmere goats is divided into the following three stages: anagen (from April to November), catagen (from December to January), and telogen (from February to March; Millar, 2002; Watabe et al., 2014; Messenger and Botchkareva, 2017). Studies by Zhang (2020) have shown that primary follicles and secondary follicles have different degrees of periodic changes throughout the year, with primary follicles being the last to grow during telogen. However, the cycle division was not completely consistent, and the activity of secondary follicles was the highest in October. November may be the transition stage of secondary follicles from anagen to catagen; January is the transition period of secondary follicles from catagen to telogen, and the follicles are still active, which can promote the growth of cashmere and finally the transition from telogen to anagen.

The growth of cashmere is affected by many factors, such as sunlight time, melatonin (MT), nutrition, genetics, and endocrine factors. The duration of direct sunlight directly affects the periodic growth of cashmere. As sunlight decreases, cashmere begins to emerge from the skin surface. As the light cycle becomes longer, the hairs fall off, and apoptosis occurs (Basheer and Abdulbari, 2018). In addition, the duration of direct sunlight affects the growth rate and yield of cashmere. The growth cycle of cashmere is also affected by the changes in light intensity. Some earlier assays have shown that with the increase in light intensity in summer, there is excessive secondary hair follicle growth from telogen to anagen, and the activity peaks in anagen. Another study showed that the length of Henderson illumination directly affected the growth of new trichomes but had no effect on the shedding of old trichomes (Salehian et al., 2015).

As an indole hormone, MT is mainly secreted by the pineal gland of the brain in humans and other mammals. Its secretion is affected by light and shows obvious diurnal changes. MT is found in the pineal gland and various other tissues and organs of vertebrates, including multifunctional molecules synthesized by the retina (Zawilska et al., 1995), bone marrow (Tan et al., 1999), gastrointestinal tract (Lepage et al., 2005), placenta (Lanoix et al., 2008), testis (Ji et al., 2012), and ovary (Tamura et al., 2017). Among its various physiological functions, MT exerts its powerful antioxidant capacity by directly scavenging free radicals and stimulating the activity of antioxidant enzymes (Reiter et al., 2017; Yang et al., 2020). In a study on the effects of light and MT implantation on cashmere production performance and regulation in Inner Mongolia white cashmere goats, periodic changes in light made the norepinephrine content periodic through the sympathetic nervous system, thus making the amount of 5-hydroxytryptamine (5-ht) and MT periodic (Tan et al., 1999). MT is secreted at higher levels at night and less in the daytime. After the summer solstice, the secretion quantities of MT increase gradually at night as the sunshine becomes increasingly shorter. After the winter solstice, as the days grow longer, and the nights grow shorter, the secretion time of MT is correspondingly shortened, and the secretion quantities are also gradually decreased (Lanoix et al., 2008; Ji et al., 2012; Tamura et al., 2017).

It was reported that MT can accelerate the hair follicle reconstruction process and induce secondary hair follicle development, which results in early entry into the next cashmere growth cycle and increases cashmere yields (Duan et al., 2017; Ge and Wang, 2018; Yang X. Z., 2020; Yang C. H., 2020). According to previous studies, two MT implants during the non-fleece period can improve the cashmere yields of Inner Mongolia cashmere goats to different degrees (Yang et al., 2020). Genes play a decisive role in the process of cashmere growth. Therefore, studying the related genes regulating cashmere growth based on MT is of great significance. *Wnt10b* is a key member of the *Wnt* family and is very important for the formation and maintenance of the hair matrix. *Wnt10b* not only promotes the development of hair follicles but also participates in the entire process of hair follicle development and cashmere periodic growth (Wu P. et al., 2020). Many studies on the classic *Wnt* signaling pathway have found that the *Wnt/*β*-catenin* signaling pathway plays an important role in hair follicle development (Lin et al., 2015; Wu Z.Y. et al., 2020). However, there is no clear mechanism by which MT regulates the cashmere growth cycle, and there is no conclusion about the role of *Wnt10b* and β*-catenin* in promoting cashmere growth through MT. In order to make up for the blank of this research, we performed many experiments in this study, including implanting MT in live cashmere goats, measuring the growth length of cashmere, observing the haematoxylin and eosin (HE) staining of skin tissues, performing Gene Ontology (GO) and Kyoto Encyclopedia of Genes and Genomes (KEGG) pathway analyses of transcript sequences of Inner Mongolia cashmere goat skins collected in February, October, and December, and using RT-PCR and western blot technology to examine the expression of the two key genes at the mRNA and protein levels. The research results provide a basis to investigate the mechanism of endogenous MT in the signaling pathway of cashmere growth in Inner Mongolia cashmere goats.

## Materials and Methods

### Sample Collection and Ethics Statement

All the animals in this study came from the Jinlai animal husbandry farm in Inner Mongolia, and the use of the cashmere goats in the study was also approved by the owner. 12 adult (2-year-old) female goats with similar body weights (∼35 kg) were selected from Inner Mongolia cashmere goats and randomly assigned to two groups of six. MT was implanted in the experimental group, while MT was not implanted in the control group.

### MT Administration

The experimental study period was 12 months, that is, during the growth cycle of cashmere. MT (provided by the special economic animal laboratory of Northeast Forestry University) was implanted subcutaneously behind the ears of cashmere goats every 2 months. Based on previous studies (Duan et al., 2015), the MT dose was 2 mg/kg live weight. The control group was not implanted, and the experiment lasted 1 year. Skin samples (1 cm × 1 cm) of the shoulder blade side were collected in February, October, and December.

### Experimental Procedures

The cashmere fiber samples were collected within 10 cm × 10 cm of the posterior part of the shoulder blade of goats. Samples were collected once a month 1 month after the start of the experiment. Each time, the sample was collected near the previous collection site on the side of the body but different from the previous collection site. Cashmere samples were collected for the determination of wool length and fineness. Skin samples of 1 cm × 1 cm on the side of the scapula of ewes were collected on the 22nd day of each month for 12 months, and the skin samples were stored in an ultralow temperature refrigerator at −80°C. Blood samples were collected immediately at 23:00 the day before each skin biopsy.

### Determination of MT Concentration in Plasma of Cashmere Goats

Blood samples were collected from the jugular vein with a 10-ml EDTA-containing vacuum tube under low red light at 12 p.m.–2 p.m. The plasma was centrifuged at 3,000 rpm/min for 10 min at 4°C and stored at −20°C until analysis. The content of MT in plasma was determined by radioimmunoassay (RIA). MT content was determined by RIA according to the method provided by the Belgian company.

### Hormone Test

Plasma MT concentrations were measured by RIA using commercial analysis equipment (Bar 3300, registered dietitian, Germany) with sensitivity and internal coefficient of variation (CV) of 2.3 pg/ml and 9.7–13.4% and 8.0–13.3%, respectively. Plasma cortisol concentrations were measured using RIA experimental tools (253 Hospital, Hohhot, Inner Mongolia), with sensitivity and internal variation coefficients (CV) of 2 ng/ml and <10% and <15%, respectively.

### Cashmere Fiber Measurement

As described by Duan et al. (2015), the wool sample contains a mixture of coarse wool and cashmere fibers that must be washed with carbon tetrachloride and warm water and then air-dried in a fume hood. The length of the cashmere fiber is measured by fixing one end of the fiber at the zero point of the ruler and gently stretching the other end until there is no bending or kinking. The fiber length of each sample was determined by the average of 100 fibers.

### Preparation of Frozen Skin Sections

The tissues were removed from −80°C, thawed at 4°C, placed in 4% paraformaldehyde solution, and fixed at 4°C overnight. The next day, the fixed tissues were washed with PBS three times for 3 min each time. After the filter paper was sucked dry, the tissues were put in 30% sucrose solution and dehydrated overnight at 4°C to the bottom of the tissues. The excess water was soaked up with filter paper, the appropriate angle was adjusted according to the section direction, the tissue was placed on the prefrozen section base, and then the tissue was embedded into the frozen section using an OTC embedding agent. The slicer was precooled 2 h in advance by setting the temperature of the apparatus at approximately −28°C, and then the tissue was sliced onto the base after it was completely frozen. Before sectioning, the sample was modified with a thickness of 20 mm. After the section was cut into the tissue, the section thickness was adjusted to 6 mm. For each section, the hair follicle shape was observed under a microscope to ensure completeness.

### HE Staining of Skin Samples Over 3 Months

Frozen sections were fixed for 1 min, washed with water for 10 s, stained with haematoxylin for 1∼2 min, and washed under running water for 15∼20 s to wash away the stain. The sections were then incubated with 1% hydrochloric acid ethanol for 1 s, washed with water for 5 s, and rinsed with water again for 15∼20 s to restore the blue coloration. Next, the sections were stained with a 0.5% eosin solution for 5∼10 s, washed for 5 s with distilled water, dehydrated with 80 and 95% anhydrous ethanol for 30 s each, and cleared with dimethylbenzene xylene for 30 s. The sections were then sealed with neutral gum, and images were acquired under a microscope.

### Determination of Relevant Parameters of Hair Follicle Groups

Images of hair follicles were taken by a microscope camera (Lycra ICC50W, Germany). Samples of sebaceous glands collected at different depths were observed with 10 fields of view each. The number of hair follicles in each counting area was calculated, plus the number of all trapped follicles at the top and left edge of the counting area. Follicles intercepted by the bottom and right margins were not included in the count.

### RNA Extraction, Library Construction, and Sequencing

In the implanted MT group and the control group, six adult female Inner Mongolia white cashmere goats with similar physical characteristics were selected. After that, skin samples of 1 cm × 1 cm from the side of the scapula were collected in February, October, and December, stored in liquid nitrogen, and transported to the laboratory for extraction of total RNA. Total RNA was extracted from skin samples using TRIzol (Life Technologies, CA, United States) according to the manufacturer’s instructions. RNA purity was checked using a NanoDrop 2000 spectrophotometer (NanoDrop Technologies, Wilmington, DE, United States), and the concentration and integrity of RNA were assessed using an Agilent 2100 Bioanalyzer and Agilent RNA 6000 Nano Kit (Agilent Technologies, CA, United States).

### Analysis of RNA-Seq Data

Approximately 4 μg of total RNA was used to prepare the RNA sequencing library using TruSeq RNA Sample Prep Kits (Illumina, San Diego, CA, United States) according to the kit’s protocol. Finally, the libraries were sequenced on an Illumina HiSeq 2500 platform using 200-bp paired-end reads.

High-quality clean reads were obtained from the raw reads by removing low-quality and adapter-contaminated reads. After that, the filtered reads were aligned to the goat (*Capra hircus*) genome27 by TopHat57. The FPKM of each gene was calculated to estimate the gene expression level (Trapnell et al., 2010). The differentially expressed genes were obtained with the standards of |fold change| >1.5 and *P*-value < 0.05.

### Pathway and Gene Enrichment Analysis

To explore the biological functions of differentially expressed genes, GO ad KEGG pathway enrichment analyses of these genes were performed. GO enrichment analyses of the differentially expressed genes were implemented by the GOseq R package. GO terms with corrected *P*-values < 0.05 were considered significantly enriched. KEGG^1^ is a database that helps users assign related molecular processes, diseases, and pathways to genes by high-throughput technology.

### Fluorescence Quantitative PCR

First, total RNA extracted was reverse transcribed into cDNA. Second, specific primers were designed by Primer 5.0 on the basis of the cDNA sequences of the goat *Wnt10b* and β*-catenin* actin genes published in NCBI and were synthesized by Shanghai Biological Engineering Co., Ltd. Next, cDNA obtained from total RNA by reverse transcription was used as a template in fluorescence quantitative PCR performed with SYBR^®^ (TaKaRa, Tokyo, Japan). Amplification of each sample by PCR was performed with 3 technical replicates.

### Western Blotting

In western blot experiments (Han et al., 2020), proteins were extracted from tissue samples in each group and quantified using the BCA Protein Assay Kit (Cat No. P0010S, Biyuntian, China). Equal amounts of proteins were subjected to SDS-PAGE and transferred to polyvinylidene fluoride membranes. Rabbit polyclonal *Wnt10b* (ab66721, 1:200, Abcam, United States) and rabbit monoclonal anti-β*-catenin* (ab17325, 1:100, Abcam, United States) antibodies were used as primary antibodies.

### Statistical Analysis

In SAS 9.0, comparisons of experimental data from two groups were performed by *t*-test, and multiple comparisons of mean values were carried out by using the Duncan method. Correlation analysis of plasma MT, cashmere growth characteristics, etc., was performed.

## Results and Analysis

### MT Concentration in Plasma of Cashmere Goats Was Significantly Increased After MT Implantation

**FIGURE 1:**
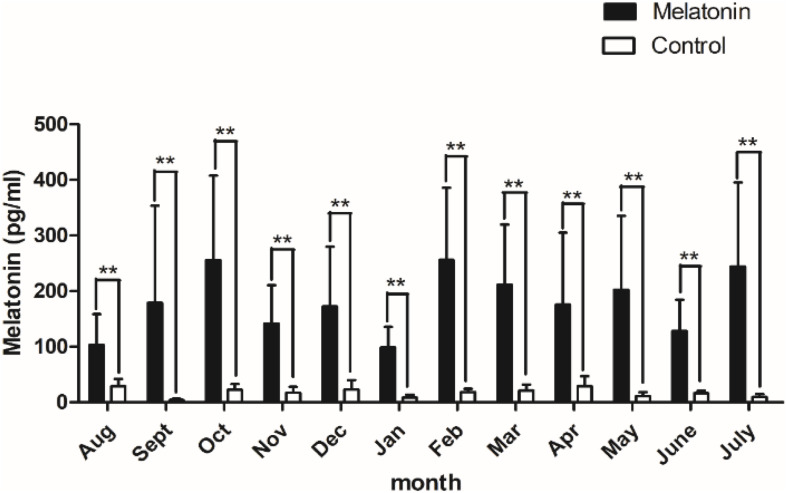
Content of melatonin in plasma. Black histogram represents experimental group and white histogram represents control group. Control: no treatment; Melatonin: melatonin implantation. Values represent means ± SD, the punctuation ** represents *P* < 0.01.

After implantation of MT, plasma MT levels increased from August to January of the following year, then began to decline after November, rose abruptly in February, and then declined gradually until June. The plasma MT content in the implanted group was approximately 10.42 times higher than that in the control group, and the MT concentration in the implanted group was significantly higher than that in the control group (*P* < 0.01; Figure 1).

### MT Can Induce the Growth of Secondary Cashmere 2 Months Earlier

**FIGURE 2:**
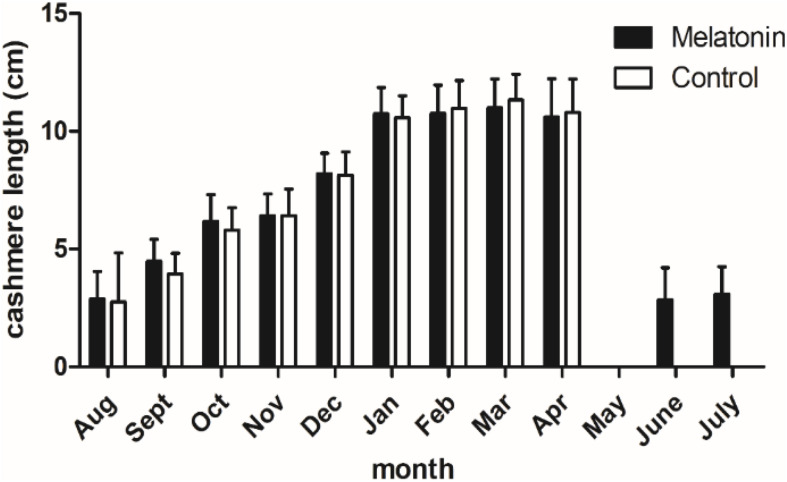
Cashmere length of the cashmere goats in a year. Black histogram represents experimental group and white histogram represents control group.

Goats in both the implanted group and the control were growing cashmere since August, and the growth basically stopped in January. From January to the cashmere period, the length of cashmere basically remains unchanged at 11 cm. Notably, in June, the MT implantation group started a new round of growth 2 months earlier than the control group, inducing secondary cashmere growth. Cashmere in the implantation group grew 3–4 cm, while no cashmere grew in the control group (Figure 2). From this experiment we found that, MT can induce the growth of secondary cashmere 2 months earlier.

### Population of Primary and Secondary Hair Follicles in Cashmere Goats During the Early Growth Period

**FIGURE 3:**
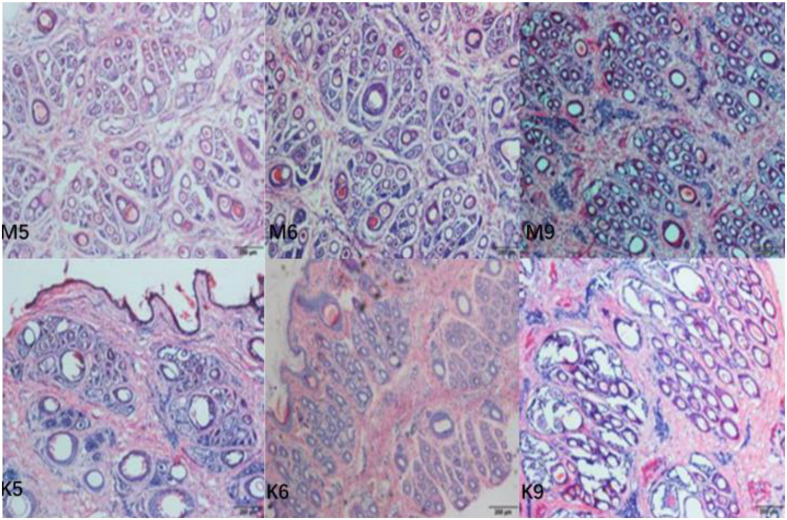
Transverse incision of skin follicles of Inner Mongolia cashmere goats in May, June, and September. M represents trial group and K represents control group. The numbers 5, 6, and 9 represent May, June, and September, respectively.

Hair follicle density (numbers of follicles per mm^2^ of skin) and S:P (ratio of secondary to primary hair follicles) were used as indicators of the population of hair follicles in the skin of cashmere goats. In the present study, goat skin tissue histological examination showed that (Figure 3) the S:P ratio in the implanted group was significantly higher than that in the control group in May and June of the pregrowth period (*P* < 0.05, Table 1). There was no significant difference in the S:P ratio between the implanted group and the control group (*P* > 0.05, Table 1) from July to the growth period (September) or the declining period (December). Therefore, we focused on cashmere goat skin tissues in the pregrowth period (May and June) and growth period (September) with significant changes in the S:P ratio. In September of the growth period, the hair follicles in the skin of the implanted group and the control group began to shrink and increase, and the S:P ratio gradually decreased (Figure 3).

**TABLE 1:** Effect of administration of melatonin to cashmere goats in one cashmere growth cycle on primary and secondary hair follicle numbers.

**Month**	**Control group**		**S:P**	**Implanted group**		**S:P**
	**PFD (n/mm^2^)**	**SFD (n/mm^2^)**		**PFD (n/mm^2^)**	**SFD (n/mm^2^)**	
January	3.37 ± 0.34	37.51 ± 1.24	11.27 ± 1.55	4.30 ± 0.16	40.42 ± 2.23	9.43 ± 0.87
February	3.83 ± 0.49	40.49 ± 2.33	10.84 ± 2.20	4.36 ± 0.10	37.96 ± 2.00	8.71 ± 0.65
March	3.88 ± 0.40	43.92 ± 1.89	11.49 ± 1.80	4.20 ± 0.53	43.02 ± 2.28	10.49 ± 1.96
April	4.06 ± 0.40	30.64 ± 2.32	7.67 ± 1.40	4.35 ± 0.51	31.96 ± 2.23	7.52 ± 1.52
May	4.46 ± 0.43	36.71 ± 2.64	8.37^a^ ± 1.51	4.58 ± 0.43	56.83 ± 1.07	12.54^b^ ± 1.46
June	4.46 ± 0.23	37.71 ± 1.55	8.49^a^ ± 0.75	4.10 ± 0.50	54.71 ± 3.77	13.56^b^ ± 2.03
July	4.75 ± 0.43	50.04 ± 3.15	10.69 ± 1.74	4.81 ± 0.53	50.00 ± 2.20	10.59 ± 1.76
August	5.06 ± 0.42	55.07 ± 2.00	10.98 ± 1.37	5.18 ± 0.42	54.70 ± 1.17	10.64 ± 1.13
September	2.84 ± 0.42	41.68 ± 1.00	15.07 ± 2.90	3.28 ± 0.36	49.35 ± 0.14	15.26 ± 1.81
October	3.40 ± 0.42	49.32 ± 2.87	14.83 ± 2.91	3.57 ± 0.44	48.45 ± 0.54	13.77 ± 1.70
November	3.13 ± 0.44	43.60 ± 0.27	14.22 ± 2.18	3.25 ± 0.32	44.00 ± 0.56	13.71 ± 1.57
December	4.04 ± 0.34	50.58 ± 2.68	12.67 ± 1.83	4.37 ± 0.46	51.62 ± 1.87	11.99 ± 1.81

*PFD, primary follicle density; SFD, secondary follicle density; S:P, ratio of secondary to primary follicles. The same letter represents no significant difference, while different letters represent significant difference in the table.*

### Screening of Differentially Expressed Genes Related to Hair Follicle Growth and Development in Cashmere Goats

**FIGURE 4:**
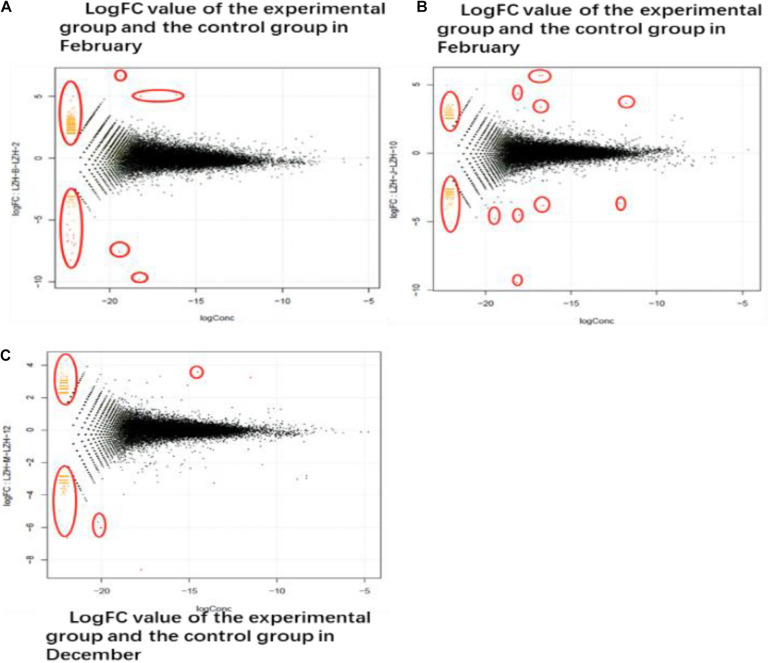
DEG different period of cashmere. **(A)** LogFC value of the experimental group and the control group in February. The transcriptome data of the experimental group and the control group in February, October, and December were screened for differentially expressed genes. The co-expressed genes were on the outside and the different genes were on the inside. **(B)** October LogFC value of the experimental group and the control group. In October, 641 genes were screened out, of which 178 were down-regulated and 463 were up-regulated. **(C)** The LogFC value of the experimental group and the control group in December. In December, 305 differentially expressed genes were screened, among which 138 up-regulated genes had 167 differences.

Differentially expressed genes were identified by comparing the transcriptome data of the experimental group and the control group in February, October, and December. As shown in Figure 4, the co-expressed genes are outside the circle, while the differentially expressed genes are inside the circle. According to the differential expression analysis, 532 differentially expressed genes were identified between the experimental group and the control group in February, among which 148 were down-regulated and 384 were up-regulated. In October, 641 differentially expressed genes were identified, among which 178 were down-regulated and 463 were up-regulated. In December, 305 differentially expressed genes were identified, among which 138 genes were down-regulated and 167 genes were up-regulated.

### Gene Functional Classification Analysis Related to Hair Follicle Growth and Development in Cashmere Goats

**FIGURE 5:**
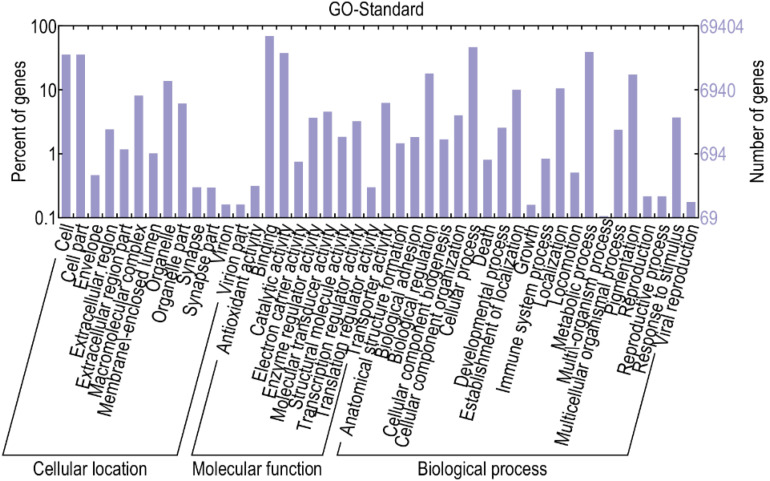
GO analysis of differential genes. This figure, clustering analysis was performed on the differentially expressed genes of the implant group and the control group in different periods. The results are summarized in three main categories: biological process, cellular component, and molecular function. The *X*-axis indicates the second level term of gene ontology; The *Y*-axis shows the number of genes.

To better understand the biological behavior of HF and skin morphogenesis, according to GO classification statistics, 44 terms were categorized into three GO categories: cell components, molecular functions, and biological processes. Among them, in the cellular component category, the top GO term was cell part. In the molecular function category, most of the terms were related to binding. In the biological process category, most of the terms were related to cellular processes (Figure 5).

Kyoto Encyclopedia of Genes and Genomes is a very useful tool in searching for genes related to metabolic or signal transduction pathways. In this study, the screened genes were annotated with the KEGG database, and the metabolic pathways of some genes were analyzed. Some of the differentially expressed genes were enriched in *Wnt*, *MAPK*, *Notch*, and other signaling pathways, which are related to hair follicle development (Zhang et al., 2020). Two different genes, *Wnt10b* and β*-catenin*, that may be related to hair follicles in these three pathways were selected for subsequent verification and analysis (Tables 2, 3).

**TABLE 2:** The primer sequences for RT-qPCR of cashmere goat gene.

**gene Gene**	**Primer sequence (5′–3′) annealing temperature the sequence of primer Tm (°C)**	**Fragment length product size**
Beta actin	F: GGCAGGTCATCACCATCGG 60	187 bp
	R: CGTGTTGGCGTAGAGGTCTTT	
Wnt10b	F: TGCTCACAACCGCAACTC 64	107 bp
	R: GGTCTCGCTCGCAGAAG	
Beta-catenin	F: GACCACAAGCAGAGTGCT 55	100 bp
	R: TGTCAGGTGAAGTCCTAAA	
SFRP1	F: GCACGACCGTGTGTCCTCCATGT 63	192 bp
	R: GCTTCTTCAGCTCCTTCTTCTTGAT	
FGF21	F: TCCCGAAAGTCTCTTGGAGC 57	110 bp
	R: ATCCGTACAGCTTCCCATCTG	
TCHHL1	F: GCCAGAAAGTGGCCCAAGATGTAT 58	192 bp
	R: CTCCAAACCATTCTCCTGTCTCAGT	

*This table shows, the specific primers were designed by Primer 5.0 according to the cDNA sequences of goat *Wnt10b*, β*-catenin*, and the actin gene with a constant expression level published in NCBI and were synthesized by Shanghai Biological Engineering Co., Ltd.*

**TABLE 3:** Genes enriched in the signal pathways related to hair follicle growth.

**Signaling pathways**	**Differential gene (ID)**	**Note**
WNT	SFRP1 (ENSBTAP00000039575)	The DOWN
	WNT10b (NM003394)	The UP
	CHP2 (ENSBTAP00000016647)	The DOWN
	Beta – catenin (NM001076141)	The UP
MAPK	FGF21 (ENSP00000222157)	The UP
	NTRK2 (ENSBTAP00000053704 – d2)	The UP
	FGF14 (ENSP00000365301)	The DOWN
	NFKB1 (ENSBTAP00000027016.1)	The UP
Notch	DLL3 (ESNP000000205143)	The DOWN
	TCHHL1 (ENSBTAP00000021074)	The DOWN

*This table, partial differential genes were enriched in *Wnt, MAPK, Notch*, and other signaling pathways. KEGG is a complete database, which serves as a bridge in major databases such as NCBI and SwissProt. The selected differential screened genes were annotated in the KEGG gene database, and some differential screened genes were analyzed in the metabolic pathway.*

### MT Promotes *Wnt10b* and β-*catenin* Gene Expression in the Skin of Inner Mongolia Cashmere Goats in Catagen and Telogen

**FIGURE 6:**
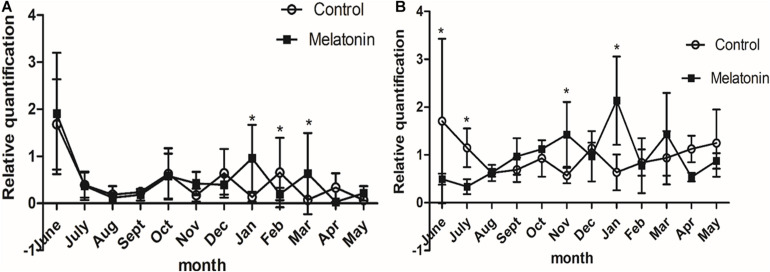
Effects of melatonin on the expression of villi growth-related genes in Inner Mongolia cashmere goats. **(A)** The relative expression quantity of the *Wnt10b* gene. The square represents the experimental group; the circle represents the control group. The *Wnt10b* gene is expressed in different degrees in each month of the year in the control group and the experimental group. Control: no treatment; Melatonin: melatonin implantation. Values represent means ± SD, *P < 0.05 vs. untreated group. **(B)** The relative expression quantity of β*-catenin* gene, the square represents experimental group; the circle represents control group. The expression level of human-catenin gene was different in each month of the year in the control group and the experimental group.

Fluorescence quantitative PCR results showed that *Wnt10b* and β*-catenin* in the control group and the experimental group were expressed to varying degrees every month of the year. The expression of *Wnt10b* was significantly increased in the catagen and telogen periods after MT implantation (*P* < 0.05), and the expression of β*-catenin* was also increased in the late anagen period and telogen period. The above two genes play important roles in the process by which MT promotes the growth of cashmere (Figures 6A,B).

### Extraction and Detection of Total RNA From Skin Tissues of Inner Mongolia Cashmere Goats

**FIGURE 7:**
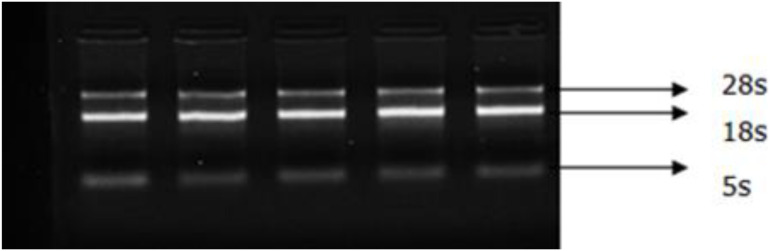
Electrophoretogram of skin total RNA. Total RNA was extracted from skin samples and detected using the NanoDrop 2000 UV spectrophotometer (Thermo Fisher). Od260/280 values were all between 1.8 and 2.0. The bands with clear integrity of 28s and 18s were detected by 1% agarose gel electrophoresis.

Total RNA was extracted from skin samples and detected by a NanoDrop 2000 UV spectrophotometer, and the products of 28S and 18S were detected by 1% agarose gel electrophoresis without protein and organic reagent contamination (Figure 7) for subsequent experiments.

### MT Had a Significant Effect on the Protein Expression of *Wnt10b* and β-*catenin* in Inner Mongolia Cashmere Goat Skin

**FIGURE 8:**
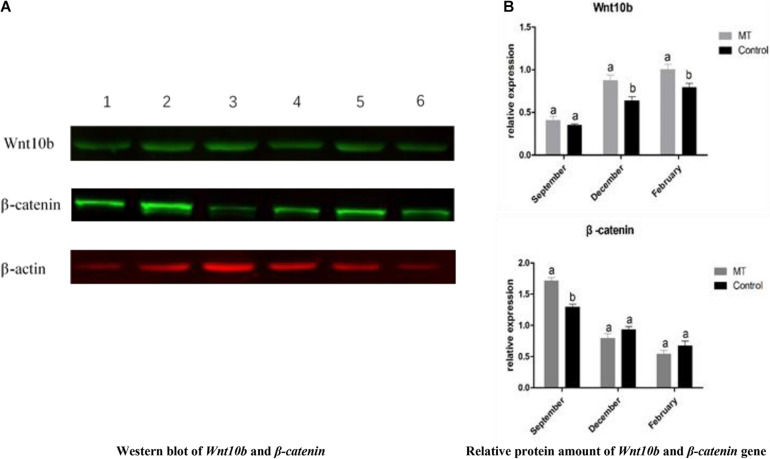
**(A)** Western blot and quantitative analysis of *Wnt10b* and β*-catenin* genes. The one, two, and three bands were *Wnt10B*,β*-catenin*, and β*-actin* of the test group in February, October, and December, respectively; the four, five, and six bands were *Wnt10B*,β*-catenin*, and β*-actin* of the control group at February, October, and December, respectively. **(B)** The same letter represents no significant difference, while different letters represent significant difference.

To investigate the underlying mechanism of MT, western blot analysis was used to detect the effects of MT implantation on the expression of the *Wnt10b* protein and β*-catenin* protein in hair follicles of cashmere goats. The results showed that the expression level of *Wnt10b* protein in the experimental group was significantly higher than that in the control group in December and February. Moreover, the expression level of the β*-catenin* protein was also significantly higher in the experimental group than in the control group in October. Taking all the data into consideration, we concluded that the *Wnt10b* protein and β*-catenin* protein play a key role in the start-up and growth of hair follicles (Figure 8). MT regulates the periodic growth of cashmere by upregulating the expression of *Wnt10b* and β*-catenin* in Inner Mongolia cashmere goats.

## Discussion

The results to date clearly support our hypothesis that continuous injection of MT can stimulate the development of secondary hair follicles in cashmere goats and speed up the hair follicle reconstruction process in cashmere goats. Importantly, MT has beneficial effects on the growth of secondary hair follicles throughout the life cycle, and by upregulating the expression of genes related to hair follicle development in cashmere goats, MT regulates the periodic growth of cashmere and induces cashmere to enter the growing period in advance, leading to secondary cashmere growth and increasing cashmere yields.

### MT Concentration in Plasma of Cashmere Goats in the Implanted Group and Control Group

The growth of cashmere has strong seasonal variation. The sunshine exposure time affects the cashmere growth rate and yield of cashmere goats. Under the influence of light intensity, MT concentration also undergoes obvious periodic changes. The secretion of MT has an important influence on the cashmere growth cycle. In this experiment, the content of MT in the plasma of the implanted and control groups was measured, and it was found that under natural light conditions, the content of MT in plasma of the control group at night was generally between 7 and 30 pg/ml, with an average of 17.34 pg/ml. After the implantation of MT, the content of MT in plasma was significantly increased. The data also indicated that the levels of MT in plasma vary greatly among different individuals at night. By implanting MT in Inner Mongolia cashmere goats, Yang found that the serum MT concentration in the treatment group was significantly higher than that in the control group, which was consistent with the experimental result of our study (Yang et al., 2020).

### MT Affects Cashmere Growth in Cashmere Goats

The results of this study showed that MT implantation could stimulate the growth of cashmere (Duan et al., 2016), which was consistent with previous results. In this experiment, there was no significant difference in cashmere length between the treatment group and the control group. In June and July of the next year, kids in the MT implant group and the first-year goats had grown 3–4 cm growth of cashmere, while in the control group, no hair had grown on the surface. This indicates that continuous implantation of MT can promote the early growth of cashmere, stimulating the growth of secondary hair follicles and accelerating the hair follicle reconstruction process, which is consistent with the results of Wuliji et al. (2006). In this study, it was also found that MT implantation could reactivate secondary hair follicles in the resting period and restore fleece activity, which was manifested as an increase in cashmere density, but the specific mechanism remains to be further studied.

For the growth of cashmere, whether in the implanting group or the control group, the growth of cashmere started in August and stopped in January. In this experiment, the growth time of wool was longer than that of cashmere, indicating that the activity duration of primary hair follicles was longer than that of secondary hair follicles. The effect of MT on the development of hair follicles of cashmere goats is larger.

Observing the phenotypic characteristics of hair follicles in tissue sections, the differences in hair follicle characteristics between the MT implantation group and the control group in May, June, and September were preliminarily investigated. In this study, the effect of MT on the number of primary hair follicles in Inner Mongolia cashmere goats was consistent with the experimental results of unimplanted MT, indicating that MT has no effect on the number of primary hair follicles (Duan et al., 2015). In this study, HE staining results showed that in the same growth cycle of the experimental group and the control group, the overall development of the primary and secondary hair follicles was roughly the same, but there were significant differences in specific months. First, the number of secondary hair follicles in the experimental group was significantly higher than that in the control group only in May and June throughout the growth cycle. Second, in the early growth stage, the position of hair follicles in the experimental group from the epidermal layer was significantly shallower than that in the control group based on observation of the longitudinal cut of the skin, indicating that the hair follicles in the experimental group were very strong at this time, while the hair follicles in the control group had already started to go deep into the skin. Therefore, it is speculated that the stimulation of exogenous MT may change the growth cycle of Inner Mongolia cashmere goat hair follicles in the early flourishing period, but it has no obvious effect on the stage from the growth period to the beginning of the next cashmere growth cycle. This finding is consistent with previous studies on the effect of MT on cashmere performance of Inner Mongolia alba cashmere goats (Yang et al., 2020). However, we want to know why the effect of MT implantation on hair follicle growth of cashmere goats is weakened after the growth period, which provides a new direction and idea for future research on MT.

### Identification of Differentially Expressed Genes Related to Cashmere Growth Based on Transcriptome Sequencing

Through transcriptome sequencing, we can determine the effect of MT implantation on the gene expression level in skin and hair follicle growth in cashmere goats at different stages. The growth cycle and formation of hair follicles are regulated by molecules and some complex signaling pathways. Some of the signaling pathways function by inhibiting or promoting factors related to hair follicle development, which are eventually transmitted to hair follicle development-related tissues to achieve whole-cycle changes in hair follicles. Therefore, finding regulatory signaling molecules at different developmental stages is the key to studying the growth and development of whole hair follicles. In this study, statistical analysis of differential gene expression showed that the most differentially expressed genes were identified in October, while the least differentially expressed genes were identified in December, indicating that gene expression was up-regulated in the initial stage of secondary hair follicle growth. In general, the activity of secondary hair follicles was the highest in October, which was also confirmed by the identification of the most up-regulated genes in October. With the progression of hair follicle growth and development, the number of up-regulated genes continued to increase, while the number of down-regulated genes began to decrease. It is worth noting that in February, hair follicle activity was lower, but a high number of up-regulated genes among the identified differentially expressed genes were found. We suspect that MT implantation may reactivate secondary hair follicles in the resting period (Wuliji et al., 2006), which is also consistent with previous research results. Finally, through GO functional classification and KEGG signaling pathway analysis, the differentially expressed genes of the experimental group and the control group in February, October, and December were enriched in biological processes, cellular components, and molecular functions. Two different genes related to hair follicular development in the *Wnt*, *MAPK*, and *Notch* signaling pathways, *Wnt10b* and β*-catenin*, were selected for further verification and analysis.

### Effect of MT Implantation on the Expression of *Wnt10b* and β-*catenin* Signaling Molecules

Reverse transcription-qPCR was used to verify the gene expression to ensure the accuracy of the study on phenotypic traits. It has been reported that there are many signaling pathways regulating the periodic growth and development of hair follicles. The *Wnt* signaling pathway is one of the central pathways (Ouji et al., 2006; Wu Z.Y. et al., 2020). *Wnt10b* and β*-catenin* are important genes related to hair follicle development in the *Wnt* signaling pathway in Inner Mongolia cashmere goats. Studies have shown that *Wnt10b* is expressed in hair matrix and inner root sheath cells and promotes the growth of hair matrix cells and hair stem lengthening. Cheng et al. (2016) showed that the expression level of Wnt10b was higher in anagen and lower in catagen and telogen. In this study, to investigate the relationship between MT and *Wnt10b* and β*-catenin* and the mechanism of the influence of MT on cashmere growth, the two genes were quantitatively analyzed by RT-qPCR. The results showed that the expression level of *Wnt10b* was significantly increased after MT implantation in catagen and telogen. In January, the hair follicle transitioned from catagen to telogen, the hair follicles shrank back under the skin, and hair follicle cells underwent apoptosis, while *Wnt10b* promoted hair matrix cell proliferation and hair stem elongation. It plays an important role in the differentiation of follicular epidermal cells (Ouji et al., 2007). Therefore, it was speculated that MT implantation increases the expression level of *Wnt10b* from catagen to telogen, which leads to the reactivation of hair follicles and the growth of cashmere 2 months earlier. *Wnt/*β*-catenin* signaling plays an important role in the growth and development of hair follicles and the proliferation and differentiation of stem cells (Chen et al., 2020). β*-catenin* has lower expression in catagen and telogen but higher expression at the end of anagen (Schmidt-Ullrich and Paus, 2005). However, the results of this study showed that MT implantation increased the expression level of β*-catenin* in late anagen and telogen. To further investigate the underlying mechanism, western blot analysis was used to detect the effect of MT implantation on the protein expression levels of *Wnt10b* and β*-catenin* in hair follicles of cashmere goats.

Previous studies have demonstrated that changes in *Wnt10b* protein expression play an important role in the growth and development of hair follicles (Li et al., 2013). The current opinion is that activation of the *Wnt10b/*β*-catenin* signaling pathway can promote hair follicle status from telogen to anagen and play a key role in the initiation of periodic hair growth. In this study, the western blot results showed that the expression level of *Wnt10b* protein was higher in the experimental group than in the control group in telogen, and the expression level of β*-catenin* protein was higher in the experimental group than in the control group in anagen. PCR results also showed that MT implantation increased the expression level of *Wnt10b* protein in telogen. Therefore, we carefully conclude that hair stromal cells were the first to pick up the signal and proliferate rapidly after MT implantation. At the same time, hair matrix cells release *Wnt10b* into the extracellular matrix and activate the *Wnt10b/*β*-catenin* pathway, which consequently alters the expression level of β*-catenin*. Thus, the β*-catenin* gene can promote the growth of hair follicle stem cells from telogen to anagen (Jones and Jomary, 2002), and the cashmere falls off and is accompanied by secondary cashmere growth.

## Summary

The present results demonstrates that MT has beneficial effects on secondary hair follicles throughout the life cycle, and by upregulating the expression of genes related to hair follicle development in cashmere goats, MT regulates the periodic growth of cashmere and induces cashmere to enter the growing period in advance.

*Wnt/*β*-catenin* signaling plays an important role in the proliferation and differentiation of stem cells by coacting on the growth and development of skin hair follicles. Western blot and PCR results showed that MT preparations increased *Wnt10b* protein expression in the regression period and the resting phase, and prompt examination of β*-catenin* protein in the hair follicle growth period was higher than that in the control group. Therefore, we cautiously conclude that after implantation of MT, hair stromal cells secrete *Wnt10b* into the extracellular matrix to activate the *Wnt10b/*β*-catenin* pathway, thereby changing the expression level of β*-catenin*.

## Data Availability Statement

The datasets presented in this study can be found in online repositories. The names of the repository/repositories and accession number(s) can be found below: NCBI (Accession: PRJNA724921, SAMN18865013–SAMN18865021).

## Ethics Statement

The animal study was reviewed and approved by the Ethics Committee of the Inner Mongolia Agricultural University. Written informed consent was obtained from the owners for the participation of their animals in this study.

## Author Contributions

YW, JiaL, and ZW: conceptualization. WG and ZeL: data curation. FZ, YJZ, and RS: formal analysis. RW, ZhL, and HX: investigation. YHZ: project administration. JinL: resources. JunL and QM: writing – original draft. All authors have read and agreed to the published version of the manuscript.

## Conflict of Interest

The authors declare that the research was conducted in the absence of any commercial or financial relationships that could be construed as a potential conflict of interest.

## Footnotes

1 https://www.kegg.jp/
